# Individual and Geospatial Determinants of Health Associated With School-Based Human Papillomavirus Immunization in Alberta: Population-Based Cohort Study

**DOI:** 10.2196/45508

**Published:** 2024-03-27

**Authors:** Jennifer Malkin, Geneviève Jessiman-Perreault, Amanda Alberga Machado, Gary Teare, Joanne Snider, Syed Farhan Tirmizi, Erik Youngson, Ting Wang, Jessica Law, Thilina Bandara, Mika Rathwell, Cordell Neudorf, Lisa Allen Scott

**Affiliations:** 1 Cancer Prevention and Screening Innovation Provincial Population and Public Health Alberta Health Services Calgary, AB Canada; 2 Communicable Disease Control Provincial Population and Public Health Alberta Health Services Edmonton, AB Canada; 3 Provincial Research Data Services Alberta Health Services Edmonton, AB Canada; 4 School of Public Health University of Saskatchewan Saskatoon, SK Canada; 5 Urban Public Health Network Ottawa, ON Canada

**Keywords:** co-design, geospatial, human papillomavirus, immunization, population-based, vaccine

## Abstract

**Background:**

Human papillomavirus (HPV) infection causes nearly all cervical cancer cases and is a cause of anogenital and oropharyngeal cancers. The incidence of HPV-associated cancers is inequitable, with an increased burden on marginalized groups in high-income countries. Understanding how immunization status varies by material and social deprivation, health system, and geospatial factors is valuable for prioritizing and planning HPV immunization interventions.

**Objective:**

The objective of this study was to describe school-based HPV immunization rates by individual and geospatial determinants of health in Alberta, Canada.

**Methods:**

Health administrative data for male and female individuals born in 2004 in Alberta were used to determine HPV immunization status based on age and the number of doses administered in schools during the 2014/2015-2018/2019 school years. Immunization status and its relationship with material and social deprivation and health system factors were assessed by a logistic regression model. Geospatial clustering was assessed using Getis-Ord Gi* hot spot analysis. Mean scores of material and social deprivation and health system factors were compared between hot and cold spots without full HPV immunization using independent samples *t* tests. A multidisciplinary team comprising researchers and knowledge users formed a co-design team to design the study protocol and review the study results.

**Results:**

The cohort consisted of 45,207 youths. In the adjusted model, the odds of those who did not see their general practitioner (GP) within 3 years before turning 10 years old and not being fully immunized were 1.965 times higher (95% CI 1.855-2.080) than those who did see their GP. The odds of health system users with health conditions and health system nonusers not being fully immunized were 1.092 (95% CI 1.006-1.185) and 1.831 (95% CI 1.678-1.998) times higher, respectively, than health system users without health conditions. The odds of those who lived in areas with the most material and social deprivation not being fully immunized were 1.287 (95% CI 1.200-1.381) and 1.099 (95% CI 1.029-1.174) times higher, respectively, than those who lived in areas with the least deprivation. The odds of those who lived in rural areas not being fully immunized were 1.428 times higher (95% CI 1.359-1.501) than those who lived in urban areas. Significant hot spot clusters of individuals without full HPV immunization exist in rural locations on the northern and eastern regions of Alberta. Hot spots had significantly worse mean material deprivation scores (*P*=.008) and fewer GP visits (*P*=.001) than cold spots.

**Conclusions:**

Findings suggest that material and social deprivation, health system access, and rural residency impact HPV immunization. Such factors should be considered by public health professionals in other jurisdictions and will be used by the Alberta co-design team when tailoring programs to increase HPV vaccine uptake in priority populations and regions.

## Introduction

### Background Information

Human papillomavirus (HPV) is the most common sexually transmitted agent in Canada, with upwards of 75% of Canadians experiencing at least 1 infection in their lifetime [1]. While most infections resolve on their own, some infections cause cell abnormalities, which can lead to cancer [2]. Infection with HPV causes nearly all cases of cervical cancer and is a cause of anogenital and oropharyngeal cancers in both men and women [3]. In Canada, cervical cancer is the 13th most common cancer diagnosed in women and the third most common among women aged 20-44 years [4].

Cervical cancer tends to be a disease of inequity, with an increased burden in high-income countries observed in disadvantaged groups [1,5]. A meta-analysis of 57 studies found that the relative risk of cervical dysplasia and invasive cervical cancer increases with decreasing socioeconomic status, with the relationship strongest in low- to middle-income countries and in North America [6]. The authors postulate that these results are due to relationships between socioeconomic status, HPV exposure, and access to cervical cancer screening programs. In addition to socioeconomic status, studies from Canada and the United States have found higher cervical cancer incidence among women living in rural rather than urban areas, which could be related to health care access [7,8]. HPV-associated cancer incidence can be addressed by increasing the uptake of the HPV vaccine, a safe and effective vaccine available since 2006 [9], but there is a need to explore factors associated with the uptake of the vaccine to close the gap in the inequities of cervical cancer incidence.

In 2018, the World Health Organization announced a global call to action for the elimination of cervical cancer, with a global strategy involving improved immunization, screening, and treatment [10]. The Canadian Partnership Against Cancer (CPAC) developed a corresponding action plan, with the immunization priority targeting having 90% of 17-year-olds in Canada fully immunized with the HPV vaccine by 2025 [11]. If this target is achieved, we could expect to see a 23% reduction in cervical cancer cases and a 21% decrease in cervical cancer–related mortalities [12]. Despite the HPV vaccine being publicly available for school-aged children, HPV coverage rates across most of Canada remain below the 90% target [1]. To address this coverage gap, it is imperative that we identify individual and geospatial determinants of health associated with being underimmunized with the HPV vaccine so that public health professionals can tailor public health planning and interventions toward priority populations.

### Objective

The primary objective of this study was to describe school-based HPV immunization rates by individual and geospatial determinants of health in Alberta. Factors were selected through an equity-informed lens to appropriately describe HPV immunization coverage by the determinants of health. To achieve this objective, the study had 2 aims:

1. To understand variation in HPV immunization uptake in relation to determinants of health within individual and geographic area-level analyses.
2. To identify clusters of regions with a higher percent of individuals without full HPV immunization (hot spots) and those with a lower percent of individuals (cold spots) and compare the distribution of individual determinants of health across those regions.

## Methods

### Setting

This study took place in Alberta, Canada, which has a fully integrated, publicly funded health care system that delivers care to nearly 4.4 million people [13]. Alberta is separated into 5 health zones based on population, geography, and the distribution of health services: Calgary, Central, Edmonton, North, and South. This study investigated Alberta’s school-based HPV immunization program, which includes HPV vaccines administered by public health in schools and in Well Child Clinics across the province [14].

**Figure 1:**
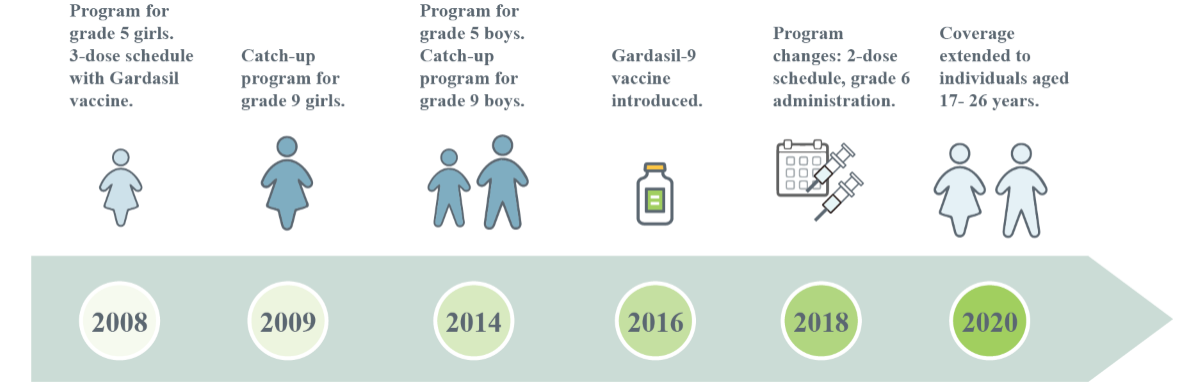
Evolution of Alberta’s human papillomavirus immunization program.

In Alberta, the Gardasil vaccine was originally offered to female individuals in grade 5 in 2008 (Figure 1 [15]) [16]. The original schedule consisted of 3 doses administered over a 6-month period. In 2014, the program was extended to include grade 5 male individuals, and in 2016, the Gardasil vaccine was replaced with the Gardasil-9 vaccine. In 2018, a total of two policy changes were implemented to the school-based program: (1) administration was pushed from grade 5 to 6, and (2) the number of doses was decreased from 3 to 2 for immunocompetent individuals aged 14 years and younger. As of 2020, all residents aged 9-26 years are eligible to receive the HPV vaccine free of charge.

### Participants

This was a cohort study looking at male and female individuals born in 2004 in Alberta. The index event was the date on which the individual turned 10 years old, with the assumption that they were in grade 5 during the 2014-2015 school year. Individuals who were 9 years old in September-December 2014 were also included in the cohort. The cohort was followed up for 5 years after their index date, from the 2014-2015 school year when they would have first become eligible to receive the HPV vaccine in grade 5, to the 2018-2019 school year when they would have been in grade 9 and reoffered the HPV vaccine in school if they hadn’t previously declined and were not up to date.

The exclusion criteria for this study included: (1) missing age, sex, or postal code (n=0); (2) not having valid Alberta health care coverage 3 years before index (necessary for complete covariate data), at index, or during follow-up (including deaths and emigration; n=8381); and (3) living outside of Alberta (n=144).

### Outcome

The outcome of interest was whether individuals in the cohort were fully or not fully immunized with the HPV vaccine. Doses were only counted toward immunization status if they occurred during the follow-up period (2014-2015 to 2018-2019 school years). Immunization status was based on age and the number of doses administered. Individuals were assessed as fully immunized after 2 doses for those 14 years of age and younger and after 3 doses for those 15 years of age and older. Individuals were considered not fully immunized if there was no record of them being immunized over the follow-up period, or they received 1 dose at 14 years of age and younger, or 1-2 doses at 15 years of age and older (ie, partially immunized). Individuals who were partially immunized were combined with those who were not immunized during the follow-up period, as they only represented 1.65% (n=746) of the cohort, and the objective of the study was to focus on those not fully immunized to inform public health planning.

Since 2008, individuals were assessed as fully immunized after 3 doses for all individuals regardless of age; however, in September 2018, the number of doses was reduced to 2 doses for those 14 years of age and younger who are immunocompetent and not infected with HIV [16]. The dose number was reduced for this group after a clinical trial found that both the 2- and 3-dose schedules produced similar vaccine efficacy [17]. Immune status was not taken into consideration for immunization status in this study, as we were not able to accurately ascertain immunocompetency or HIV status from health codes associated with the Canadian Institute for Health Information (CIHI) grouper. Immunization status for this study was classified based on post-2018 age and dose number standards throughout the entire follow-up period.

In order to achieve an optimal immune response, the first and last dose of either a 2- or 3-dose HPV vaccine series is meant to be administered over a 6-month interval [4]. The time between the first and last dose was not considered in this analysis as we deviated from the historical vaccine schedule and instead based immunization status on post-2018 age and dose number standards (ie, individuals were assessed as fully immunized at 2 doses for those 14 years of age and younger, or 3 doses for those 15 years of age and older).

### Data Sources

**Figure 2:**
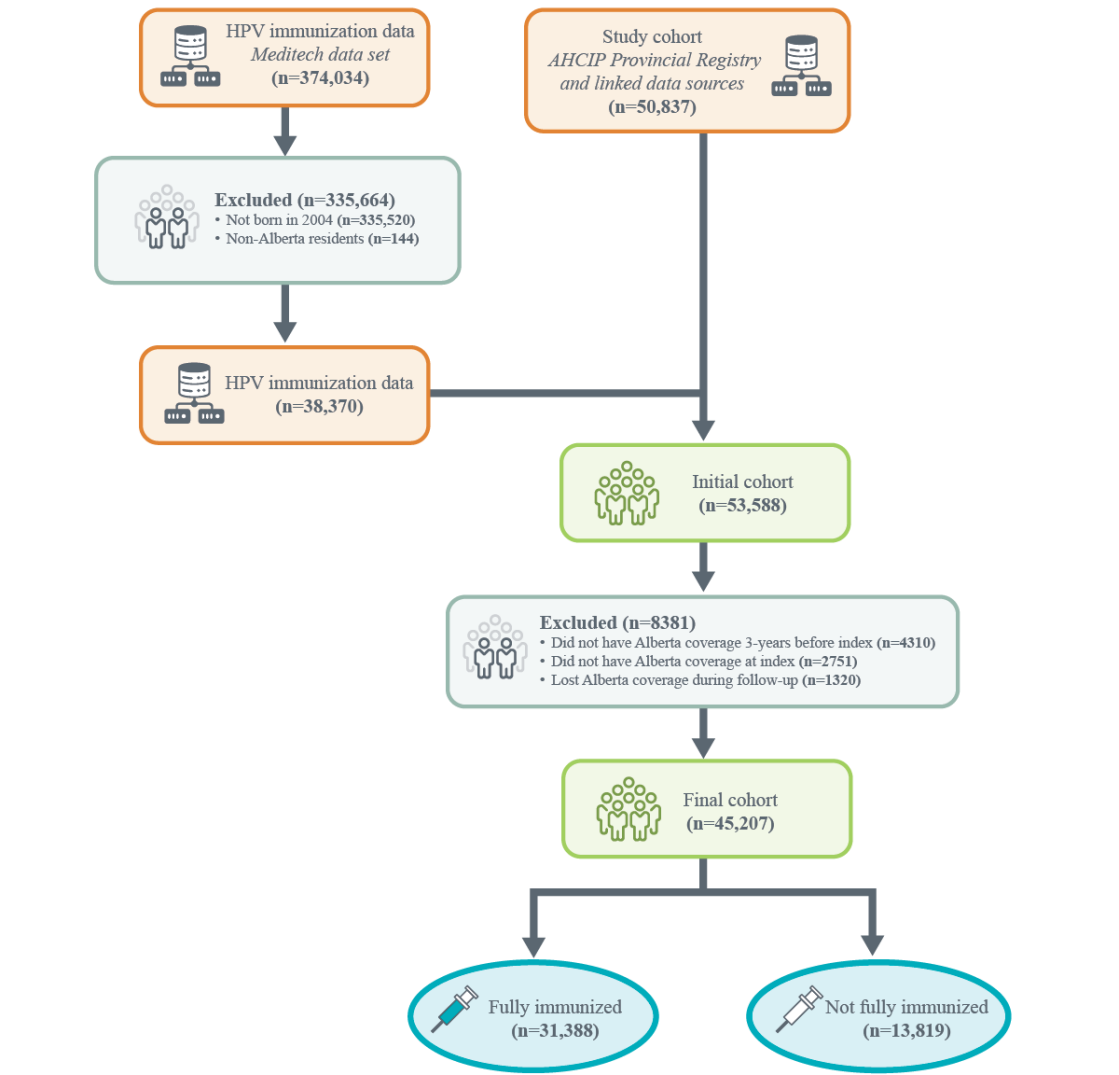
Creation of the study cohort data set. The (n=2751) surplus in the initial cohort when compared to the original Alberta Health Care Insurance Plan (AHCIP) Provincial Registry and linked data sources study cohort was due to the “did not have Alberta coverage at index” exclusion criterion being pre-applied to the AHCIP Provincial Registry but not the Meditech data set. This surplus is removed in the next exclusion step. HPV: Human Papillomavirus.

The study cohort was created using administrative data extracted from the Alberta Health Services Enterprise Data Warehouse. The Alberta Health Care Insurance Plan (AHCIP) Provincial Registry contains population demographics for all persons in Alberta covered for basic medical and hospital insurance during a given fiscal year and was used to define our study cohort. Using a provincial health care number, the cohort was linked to other population-based data sets, including the Alberta Health Postal Code Translator File, the 2016 Pampalon Index, physician claims, the Discharge Abstract Database (DAD), and the National Ambulatory Care Reporting System (NACRS). The Alberta Health Postal Code Translator File was used to establish geographical boundaries to classify local geographic areas (LGAs), health zones, and urban and rural areas. The 2016 Pampalon Index uses Census data at the dissemination area level to present socioeconomic disparities among the population. The index is made up of material and social components, stratifying the population into 5 quintiles, with the first level being the least deprived, and the fifth being the most deprived. The material component is characterized by low income, an insecure job situation, and low education, while the social component is defined by being a single parent, being separated, divorced, or widowed, and living alone [18]. Physician claims capture data for fee-for-service and shadow-billed (ie, bills submitted for reporting purposes, but practitioners compensated through alternate methods) claims by physicians and provides information on service dates, number of visits, and services provided [19]. Physician claims data cover the 3 years before index (ie, the date an individual turned 10 years old). DAD and NACRS capture acute care hospitalizations and community-based and hospital-based ambulatory care, including emergency department visits, respectively, in Alberta, and include records for demographic, diagnostic, procedural, and treatment information for each visit [20]. DAD and NACRS were used to define the CIHI grouper, which categorizes individuals into health profile groups based on the history of health services received and the presence of health conditions. Individuals from the study cohort were assigned a health profile group based on diagnoses from a variety of health sectors, including but not limited to hospitals, emergency rooms, urgent care centers, and specialist and family practice settings. A total of 226 health conditions from 16 categories (eg, minor acute, major chronic, and other mental health) were used to synthesize an individual’s clinical profile to the most complex and clinically relevant health conditions present. Youths were placed into 1 of the following 3 categories: health system nonuser, health system user without health conditions (ie, without 1 of the 226 health conditions), and health system user with health conditions (ie, with 1 of the 226 health conditions). Groups were based on data from the 2013-2014 fiscal year [20,21]. The data set was then merged with the Meditech Health Information System, which contains information on all HPV vaccines administered by public health in schools and in Well Child Clinics across the province. After merging the Meditech Health Information System data set with the AHCIP Provincial Registry and linked population-based data sources and applying the exclusion criteria, the final merged data set consisted of 45,094 observations (Figure 2).

Individual postal code was geocoded using the Alberta Health Postal Code Translator File and estimates of percent of individuals without full HPV immunization were aggregated at the LGA. The LGAs were chosen for this spatial analysis as they are the smallest geographic area that Alberta Health Services and Alberta Health use as official geographies [22], and they determine how health services are distributed across Alberta. In Alberta, there are 132 LGAs of varying sizes divided across the 5 health zones.

### Analysis

**Figure 3:**
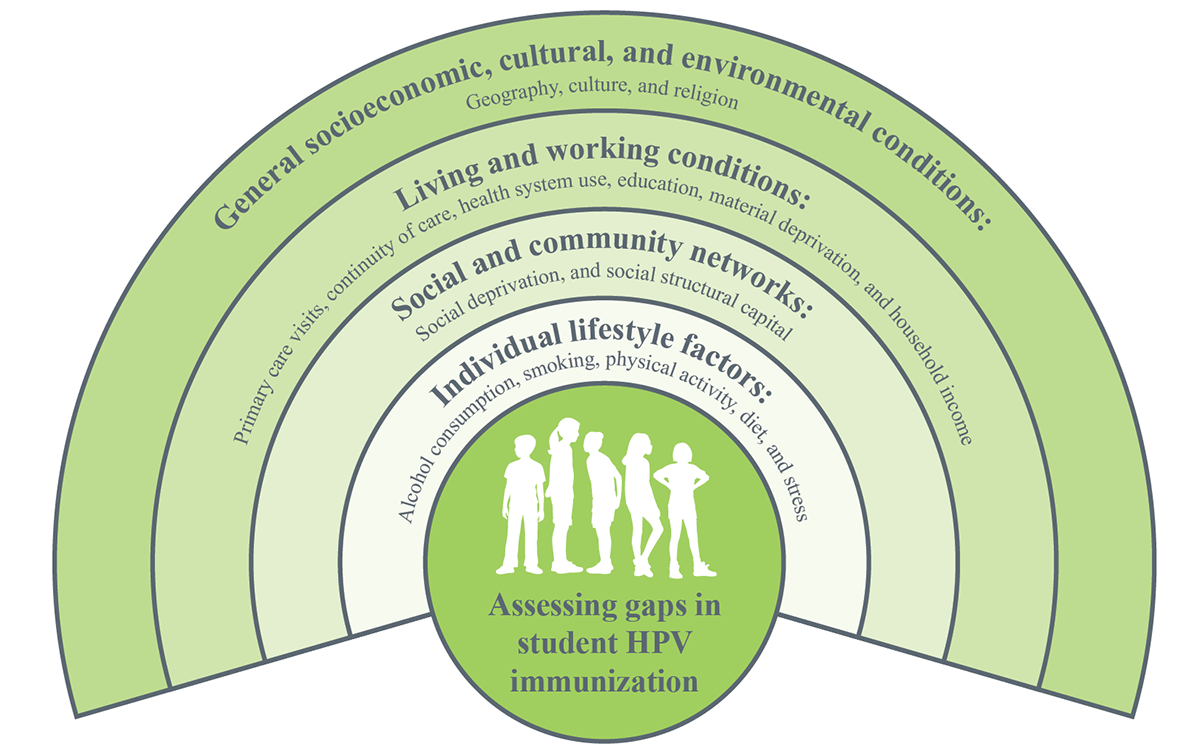
Conceptual framework of determinants of health that impact student human papillomavirus (HPV) immunization.

Variation in HPV immunization status and its relationship with determinants of health were assessed by analytical, data visualization, and geospatial techniques. Variables for inclusion in this analysis were selected a priori based on our conceptual framework (Figure 3; adapted from Dahlgren and Whitehead [23]) and data availability. Our conceptual framework was based on determinants of health documented in the literature (for example [5,24-28]) that impact student HPV immunization coverage.

Characteristics were summarized by HPV immunization status using counts and percentages. The use of mean and SD was determined based on the distribution of the data. Before their inclusion in the logistic regression analysis, correlations between covariates were checked using chi-square and Spearman rank-order correlation tests, where appropriate. A logistic regression model was used to estimate the odds ratios (ORs) comparing not fully immunized with fully immunized status for the characteristics of interest. ORs, adjusted odds ratios (aORs), and 95% CIs are reported. Observations were excluded from the logistic regression if they had missing covariate values (n=1694). No variables had more than 5% missing observations.

Global Moran’s *I* was used to detect whether spatial autocorrelation exists, meaning that individuals without full HPV immunization are spatially clustered in Alberta. Moran’s *I* ranges from +1 to –1 with positive values indicating spatial autocorrelation (ie, clustering of similar rates), while negative values indicate similar rates are located far away from each other (ie, dispersion). A *P* value that is smaller than .05 means we can reject the null hypothesis of complete spatial randomness and accept that spatial autocorrelation exists and there is clustering [29].

To detect the location and magnitude of spatial clustering, we used local Getis-Ord Gi* optimized hot-spot analysis. Getis-Ord Gi* hot-spot analysis was used to identify areas of statistically significant clusters of LGAs with high rates of individuals without full HPV immunization (ie, hot spots) and statistically significant clusters of LGAs with low rates of individuals without full HPV immunization (ie, cold spots). We used optimized hot-spot analysis to generate a spatial weight matrix. The optimal fixed distance threshold band was approximately 110 kilometers. Each geographic unit had on average 5 nearest neighbors, and 43.1% (56/132) of geographic units had less than 8 nearest neighbors. One geographic unit was considered an outlier and not used to calculate the spatial weight matrix. Due to a sample size of less than 20 cases in 16 LGAs, data have been suppressed in the hot spot analysis. All spatial analysis was conducted using ArcGIS Pro (version 2.6.3; Esri). Finally, independent sample *t* tests were used to compare whether material and social deprivation and the number of visits to a general practitioner (GP) varied between hot spots of individuals without full HPV immunization and cold spots.

### Ethical Considerations

This study received ethics approval by the Health Research Ethics Board of Alberta (HREBA.CC-20-0425).

### Co-Design Team

A multidisciplinary team based in Alberta formed a co-design team comprising experts in cancer prevention, epidemiology, immunization programming, biostatistics, public health planning, and communicable disease control to collaborate on this study. Co-design, or collaborative design, involves the active engagement of stakeholders involved with an issue who work together to design solutions to address the problem [30,31]. By drawing on the collective perspectives, experiences, and strengths of the group, there can be improved idea generation, better interventions, and enhanced outcomes [30]. The co-design team designed the study protocol, reviewed the study results, and will use the findings to identify priority populations and regions that should be engaged when designing interventions to improve HPV immunization coverage across Alberta. This collaboration facilitated the creation of relevant questions, appropriate methods, and how the results could be used for public health planning.

## Results

### Descriptive Statistics

Descriptive statistics summarizing characteristics by HPV immunization status are displayed in Table 1. While the majority of youths who were fully immunized received their doses in grade 5, nearly 10% (2750/31,388) of youths received all of their doses in grade 9. A lesser proportion of youths who were fully immunized were from the Central, North, and South zones, compared to those in the Calgary and Edmonton zones. Those who were fully immunized tended to live in urban rather than rural areas. Youths who were not fully immunized tended to live in areas with more material and social deprivation than those who were fully immunized. Youths who were fully immunized were more likely to have had a GP visit in the 3 years before the index and tended to have a higher number of GP visits compared with youths who were not fully immunized. Those who were not fully immunized were more likely to be health system nonusers or health system users with health conditions.

**Table 1:** Characteristics of Albertans by human papillomavirus immunization status (n=45,207).

Characteristic		Fully immunized (n=31,388)	Not fully immunized (n=13,819)
**Sex, n (%)**			
	Female	14,924 (47.55)	7187 (52.01)
	Male	16,464 (52.45)	6632 (47.99)
**Grade received, n (%)**			
	Grade 5	27,632 (88.03)	211 (28.28)
	Grade 9	2750 (8.76)	474 (63.54)
	Both	823 (2.62)	0 (0)
	Neither	183 (0.58)	61 (8.18)
	Not applicable^a^	0 (0)	13,073 (94.6)
**Health zone, n (%)**			
	Calgary	12,365 (39.39)	4352 (31.49)
	Central	3389 (10.80)	2153 (15.58)
	Edmonton	9600 (30.58)	3815 (27.61)
	North	3633 (11.57)	2252 (16.30)
	South	2401 (7.65)	1247 (9.02)
**Area, n (%)**			
	Urban	24,593 (78.35)	9531 (68.97)
	Rural	6795 (21.65)	4288 (31.03)
**Material deprivation, n (%)**			
	1 (least deprived)	4995 (16.46)	1810 (13.74)
	2	5895 (19.43)	2312 (17.55)
	3	5914 (19.49)	2511 (19.06)
	4	6725 (22.16)	2893 (21.96)
	5 (most deprived)	6812 (22.45)	3646 (27.68)
**Social deprivation, n (%)**			
	1 (least deprived)	6797 (22.40)	2717 (20.63)
	2	5386 (17.75)	2167 (16.45)
	3	5974 (19.69)	2540 (19.28)
	4	6452 (21.26)	2906 (22.06)
	5 (most deprived)	5732 (18.89)	2842 (21.58)
**GP^b^ Visits (3 years before index), n (%)**			
	Yes	27,259 (86.85)	9813 (71.01)
	No	4129 (13.15)	4006 (28.99)
Number of GP visits (3 years before index), mean (SD)		5.92 (5.42)	5.19 (4.86)
**CIHI^c^ grouper, n (%)**			
	Health system nonuser	7343 (23.39)	5641 (40.82)
	Health system user with no health conditions	2889 (9.20)	907 (6.56)
	Health system user with health conditions	21,156 (67.40)	7271 (52.62)

^a^No school grade dose data are available for these records as these are youths who did receive a human papillomavirus dose from public health.

^b^GP: general practitioner.

^c^CIHI: Canadian Institute for Health Information.

### Logistic Regression

A logistic regression model was fitted to estimate the aORs of not being fully HPV immunized, compared to being fully immunized according to study characteristics (Table 2). The odds of those who did not see their GP in the 3 years before index not being fully immunized were 1.965 (95% CI 1.855-2.080) times higher than those who did see their GP. The odds of health system users with health conditions and health system nonusers not being fully immunized were 1.092 (95% CI 1.006-1.185) and 1.831 (95% CI 1.678-1.998) times higher than health system users without health conditions. The odds of those who lived in areas with the most material and social deprivation not being fully immunized were 1.287 (95% CI 1.200-1.381) and 1.099 (95% CI 1.029-1.174) times higher than those who live in areas with the least deprivation. The odds of those who lived in rural areas not being fully immunized were 1.428 (95% CI 1.359-1.501) times higher than those who lived in urban areas.

**Table 2:** Logistic regression model examining the odds of not being fully immunized based on determinants of health.

Characteristic		Odds of not being fully immunized	
		OR^a^ (95% CI)	aOR^b^ (95% CI)
**GP^c^ visits**			
	Yes	1.00 (ref^d^)	1.00 (ref)
	No	2.695 (2.565-2.831)	1.965 (1.855-2.080)
**CIHI^e^ grouper**			
	Health system user without health conditions	1.00 (ref)	1.00 (ref)
	Health system user with health conditions	1.095 (1.011-1.185)	1.092 (1.006-1.185)
	Health system nonuser	2.447 (2.254-2.657)	1.831 (1.678-1.998)
**Material deprivation**			
	1 (least deprived)	1.00 (ref)	1.00 (ref)
	2	1.082 (1.007-1.163)	1.054 (0.978-1.135)
	3	1.172 (1.091-1.258)	1.110 (1.031-1.195)
	4	1.187 (1.108-1.272)	1.071 (0.996-1.151)
	5 (most deprived)	1.477 (1.381-1.580)	1.287 (1.200-1.381)
**Social deprivation**			
	1 (least deprived)	1.00 (ref)	1.00 (ref)
	2	1.007 (0.941-1.076)	1.029 (0.960-1.102)
	3	1.064 (0.997-1.134)	1.036 (0.969-1.107)
	4	1.127 (1.059-1.199)	1.048 (0.982-1.118)
	5 (most deprived)	1.240 (1.164-1.321)	1.099 (1.029-1.174)
**Area**			
	Urban	1.00 (ref)	1.00 (ref)
	Rural	1.628 (1.557-1.703)	1.428 (1.359-1.501)

^a^OR: odds ratio.

^b^aOR: adjusted odds ratio.

^c^GP: general practitioner.

^d^ref: reference group.

^e^CIHI: Canadian Institute for Health Information.

### Geospatial Analysis

Spatial autocorrelation of individuals without full HPV immunization at the LGA level was detected using Global Moran’s *I*. With a Moran’s *I* index value of 0.051, a *z*-score of 3.74, and *P*<.001, the null hypothesis was rejected, and it was concluded that there was more than a 99% probability that the distribution of individuals without full HPV immunization formed a clustered pattern by LGA in Alberta, Canada.

**Figure 4:**
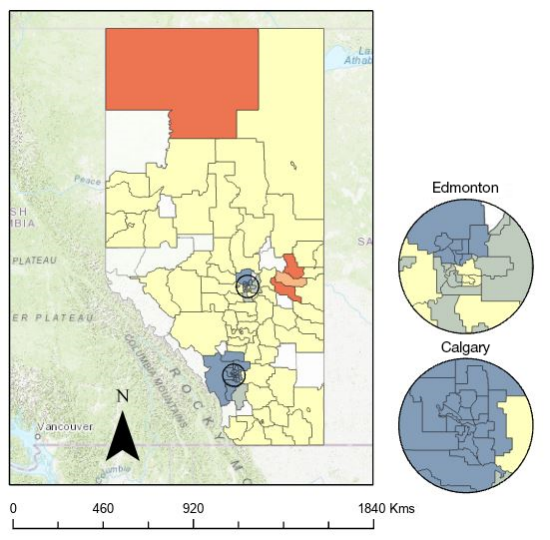
Areas of statistically significant clusters of local geographic areas with high (ie, hot spots) and low (ie, cold spots) rates of male and female individuals born in 2004 without full human papillomavirus immunization in Alberta, Canada.

Getis-Ord Gi* hot-spot analysis was used to determine the location and magnitude of the spatial autocorrelation. Results from Figure 4 indicate that there were 48 statistically significant hot or cold spots of individuals who were not fully immunized with the HPV vaccine. There were 2 clusters of hot spots in the North zone (High Level LGA) and on the eastern side of Alberta that cross the North and Central zones (St. Paul, Two Hills County, and Vegreville/Minburn County LGAs). There were also 2 large statistically significant cold spots within the Edmonton and Calgary zones. Nonsignificant data are presented in yellow. Due to small sample sizes (ie, under 20 individuals making up the estimate), data in 16 LGAs were suppressed—these areas are presented in white.

Independent samples *t* tests were used to determine if determinants of health vary between hot spots of individuals without full HPV immunization and cold spots (Table 3). Individuals living in hot spots were more likely to have a higher mean material deprivation score than those living in cold spots. Individuals living in hot spots had a statistically lower mean number of GP visits compared to those living in cold spots.

**Table 3:** Descriptive statistics and independent samples *t* test comparisons of material and social deprivation indicators and health system use between hot and cold spots of youths not being fully immunized with the human papillomavirus vaccine in Alberta.

Variable	Hot spot, mean (SD)	Cold spot, mean (SD)	*P* value
Material deprivation	4.30 (0.14)	2.91 (0.99)	.008
Social deprivation	3.15 (1.08)	3.06 (0.90)	.85
Number of GP^a^ visits	2.78 (1.04)	4.84 (1.15)	.001

^a^GP: general practitioner.

## Discussion

### Principal Results

This population-based cohort study analyzed material and social deprivation, health system, and geospatial factors to describe school-based HPV immunization rates in Alberta. Overall, 69% (31,388/45,207) of the cohort was fully immunized with the HPV vaccine, and 31% (13,819/45,207) were not fully immunized. One interesting finding was that while most fully immunized youths in Alberta received their doses in grade 5, the grade 9 catch-up program caught almost an additional 10% (2750/31,388). While the majority of Canadian provinces and territories implemented catch-up programs for girls when the HPV vaccine was initiated in the respective jurisdiction, only 2 other provinces implemented such a program for boys [32]. Alberta continues to rescreen youths in grade 9 to determine whether they are missing one or more doses of their HPV vaccine. Considering the proportion of youths who were immunized by this catch-up program, it is an initiative that other jurisdictions could consider implementing to increase HPV vaccine uptake.

The results indicate that a lack of health system use may be an important indicator of HPV vaccine uptake. Individuals who do not visit their GP are less likely to be recommended the HPV vaccine by a health care provider, which has been demonstrated to impact vaccine acceptance, uptake, and series completion [33-39]. In urban areas, this may be more of a matter of taking advantage of missed opportunities for education when children and their parents or guardians visit their health care provider. In rural areas, it may be more of an issue of access to a health care provider [1,40]. On the other hand, the parents or guardians of health system nonusers may have lower trust in public institutions, which may impact their willingness to consent to the HPV vaccine for their child. For example, direct or intergenerational trauma associated with colonization or previous negative experience in health care, such as racism or discrimination, has discouraged trust in government-provided health care for some Indigenous Peoples of Canada, causing some individuals to avoid or delay seeking care [41]. One way we can improve the current system is by adopting an approach to cultural safety, which involves practitioners self-reflecting on their assumptions, challenging inequalities, and improving health care access by shifting the power from the practitioner to the patient [41,42].

Experiencing more material and social deprivation appears to be associated with not being fully immunized with the HPV vaccine. Other studies in Canada have found that living in areas of high material and social deprivation was associated with lower HPV vaccine refusal [43], those living in areas of high material deprivation were more likely to complete the HPV vaccine series [44], and those living in areas of high social deprivation had lower HPV vaccine coverage [45]. These results indicate that social and material deprivation as it relates to HPV vaccine uptake is complex, and individual-level associations may be difficult to discern using area-level information. Further analysis of individual-level factors should be an area of further research.

The results indicate that living in rural areas may be associated with not being fully immunized with the HPV vaccine. This is reflected in coverage variation by health zone, as a lesser proportion of youths who were not fully immunized were from the Central, North, and South health zones, with clustering occurring in the North and Central zones, which comprise more rural communities than the Calgary and Edmonton zones. With rural Canadian communities experiencing a shortage of family doctors [46], rural youths and their parents or guardians may not be receiving a recommendation to receive the HPV vaccine in school, which has been shown to influence vaccine acceptance, uptake, and series completion [33-39]. There is also an imbalance between the supply of nurses working in rural and remote areas in Alberta (12% in 2015) and the portion of the population residing there (18.5% in 2015) [47]. This discrepancy could lead to rural and remote public health nurses having less capacity to complete vaccine series for school-aged children. Parents and guardians in rural communities may also be declining the HPV vaccine for their child due to increased levels of vaccine hesitancy, but further research is needed to dissect the relationship between vaccine hesitancy and immunization behaviors at the community level [48].

### Strengths and Limitations

This study has several methodological strengths. Large population-based sample sizes have high internal validity, and findings could be generalized to jurisdictions similar to Alberta. In addition, the HPV immunization data were sourced from the Meditech Health Information System, an administrative database, which is significantly more reliable than self-reporting, which is susceptible to recall bias. Furthermore, by solely focusing on doses administered by public health, recommendations for improvement can focus on school-based programs.

In the past, it has taken an average of 17 years for research evidence to move into practice and implementation [49]. To circumvent this issue, it is critical for researchers to partake in integrated knowledge translation, wherein knowledge users (eg, policy makers, practitioners, and administrators) are collaborators throughout the entire research process [50]. By focusing on questions that are relevant to practice and working with partners who are capable of implementing identified recommendations, integrated knowledge translation can effectively promote the uptake and use of research findings [50-52]. The key strength of this study was the use of a co-design team to enable an integrated knowledge translation approach. By using an integrated knowledge translation approach, the co-design team strives to shorten the typical 17-year research evidence-to-innovation gap, integrating study findings into public health planning to reach the 90% immunization target by 2025. Notably, these results will be supplemented with information on barriers and facilitators of the school-based HPV immunization program from the perspective of parents and guardians, as well as program leaders and front-line providers, through a CPAC-funded study being conducted by members of the co-design team [15].

There are several methodological limitations that should be noted, as they will impact the generalizability of the findings. First, HPV coverage was determined by applying current immunization standards to all follow-up years, despite the policy change that occurred in 2018, and immune status was not considered when determining immunization status. While this may overestimate the number of individuals fully immunized for HPV in the pre-2018 period, the objectives of this study center around determining the characteristics associated with being underimmunized in order to inform improvements to the current school-based immunization program, including who and where to target improvements, rather than solely determining coverage rates.

Second, the cohort denominator is population-based rather than school-based due to a lack of school list availability. Consequently, the AHCIP Provincial Registry needed to be used as a proxy denominator, which could result in underestimated coverage rates.

Third, the outcome variable of not being fully immunized may not necessarily mean that the individual was not fully immunized in certain situations. Immunization data were gathered from the Meditech Health Information System, thus only allowing us to classify those HPV doses administered by Alberta public health. Children may have received immunization at a local pharmacy, family doctor, or out of province, and consequently would have been misclassified as not being fully immunized.

Fourth, the exclusion criterion of removing those who did not have valid Alberta health care coverage over the full study period represented more than 5% (8381/53,588) of the original cohort. These individuals were excluded due to incomplete data, either for covariate data before index or immunization data at index or during follow-up. Due to incomplete data, it cannot be determined if these individuals would have a different propensity to be fully immunized if they had had access to the school-based HPV immunization program throughout the entire study period; thus, these individuals were excluded to avoid bias in the results.

Fifth, attributing area-level information to individuals, as was the case for the Pampalon Index and comparisons of the hot and cold spots, assumes area-level data can be attributed to all individuals in that geographic location, which is an ecological fallacy. Research has also shown that such inferences can attenuate true relationships between the factor and the outcome (ie, immunization status) [53]. These results will have to be interpreted with caution to ensure appropriate conclusions are drawn.

Lastly, LGA was used as the boundary for our geographic mapping. This boundary can help identify areas of the province to prioritize for intervention, but LGAs are based on population patterns of health system access (ie, use of hospital services) and may not capture the similarities in HPV immunization outcomes across these boundaries. Further research could explore the use of other geographic boundaries that might provide additional context into HPV immunization rates, such as school districts. Despite this limitation, LGA is currently the smallest official geographic unit used by Alberta Health Services and Alberta Health [22], provides sufficient sample size to allow for accurate and precise estimates in most LGAs, and has been used in other geospatial studies of health outcomes in Alberta [54-56]. Furthermore, in 16 LGAs, there were fewer than 20 cases, making up the estimates for the geospatial analysis. While conducting hot-spot analysis on small sample sizes is possible, it limits the reliability of the estimates over time. Therefore, we chose to suppress the findings from these 16 LGAs, which limits the information available in those specific areas but was necessary to avoid reporting hot spots based on potentially unstable estimates.

### Conclusions

This study highlighted how HPV immunization status varies by individual and geospatial determinants of health. Those who were not fully immunized with the HPV vaccine tended to be health system nonusers, less likely to visit their GP, live in rural areas, and experience higher levels of material and social deprivation. In Alberta, there were 2 statistically significant hot spots of individuals not fully immunized with the HPV vaccine, located on the north and east sides of the province, with individuals living in hot spots being more likely to have a higher mean material deprivation score and less likely to visit a GP than those living in cold spots.

Study results will enable the co-design team to make evidence-informed decisions when tailoring the school-based HPV immunization program for priority populations and regions. Public health professionals in other jurisdictions should consider these determinants of health when designing interventions to increase HPV immunization coverage.

## Abbreviations

AHCIP: Alberta Health Care Insurance Plan
aOR: adjusted odds ratio
CIHI: Canadian Institute for Health Information
CPAC: Canadian Partnership Against Cancer
DAD: Discharge Abstract Database
GP: general practitioner
HPV: human papillomavirus
LGA: local geographic area
NACRS: National Ambulatory Care Reporting System
OR: odds ratio
